# Application of the QFD-fuzzy-SERVQUAL methodology as a quality planning tool at the surgical centre of a public teaching hospital

**DOI:** 10.1186/s12911-022-01746-4

**Published:** 2022-01-07

**Authors:** Jurandir Barreto Galdino Junior, Hélio Roberto Hékis, José Alfredo Ferreira Costa, Íon Garcia Mascarenhas de Andrade, Eric Lucas dos Santos Cabral, Wilkson Ricardo Silva Castro, Davidson Rogério de Medeiros Florentino, Tiago de Oliveira Barreto, João Florêncio da Costa Júnior

**Affiliations:** 1 Master in Production Engineering at Federal University of Rio Grande do Norte (UFRN), Natal, RN Brazil; 2 Production and Systems Engineering - Concentration Area - Business Management from the Federal University of Santa Catarina - UFSC (2004). Adjunct Professor at Federal University of Rio Grande do Norte, Natal, RN Brazil; 3 Electrical and Computer Engineering - UNICAMP (1999). Full Professor at Federal, University of Rio Grande do Norte, Natal, RN Brazil; 4 Health Sciences / Medicine from the Federal University of Rio Grande do Norte (2008). Professor at Universidade Potiguar (UnP), Natal, RN Brazil

**Keywords:** Surgical centre, Fuzzy logic, QFD, Quality, SERVQUAL

## Abstract

**Background:**

In Brazil, many public hospitals face constant problems related to high demand vis-à-vis an overall scarcity of resources, which hinders the operations of different sectors such as the surgical centre, as it is considered one of the most relevant pillars for the proper hospital functioning, due to its complexity, criticality as well as economic and social importance. Proper asset management based on well-founded decisions is, therefore, a *sine-qua-non* condition for addressing such demands. However, subjectivity and other difficulties present in decisions make the management of hospital resources a constant challenge.

**Methods:**

Thus, the present work proposes the application of a hybrid approach, formed by the QFD tools, fuzzy logic and SERVQUAL as a decision support tool for the quality planning of the surgical centre of the Onofre Lopes Teaching Hospital (*Hospital Universitário Onofre Lopes*—HUOL). To accomplish such objective, it was necessary to discover and analyse the main needs of the medical team working in the operating room, through the application of the SERVQUAL questionnaire, associated with fuzzy logic.

**Results:**

Then, the most relevant deficiencies were transformed into entries for the QFD-fuzzy, where they were translated into project requirements. Soon after, the analysis of the existing relationships between the inputs and these requirements was carried out, generating the ranking of actions with the greatest impact on the improvement of the surgical centre overall quality.

**Conclusions:**

As a result, it was found that the proposed methodology can optimize the decision process to which hospital managers are submitted, improving the surgical centre operation efficiency.

## Background

Throughout the world, health services are compelled to deliver excellent results. Even when compared to other sectors, the health sector must manage the quality of its services more strictly, as it directly affects the population's living conditions and wellbeing. The provision of quality health services, therefore, has a positive impact on the economy, which in turn benefits the population as a whole; leading to the conclusion that improving health services is an absolute priority [1].

Focusing on the Surgical Centres (SC), it is possible to argue that it is the sector with the highest costs within the hospital. According to Bidassie et al. [2] as well as Nazif [3], it is estimated that approximately 40% of hospital costs and revenues are caused by the SC. Thus, the use of quality tools, methodologies for decision support and operational research, are described in the literature as appropriate instruments to generate improvements whilst reducing costs in a SC [4].

The Brazilian state, according to the 1988 Constitution, must provide health services to the population in an appropriate manner. However, due to the various difficulties faced by the country, basic resources to ensure health for citizens are often scarce. Moreover, about 50% of the costs arising from the national health system come from hospital expenses, with the SC being the main responsible for this [5].

The Onofre Lopes University Hospital (HUOL), which is part of the Federal University of Rio Grande do Norte (UFRN) and is associated with the Brazilian Hospital Services Company (EBSERH), has 31,569.45 m^2^ of built area, 24 ICU beds, 19 adults and 5 pediatric, 242 infirmary beds and 12 operating rooms, 2 in the ophthalmology sector, 7 in the SC and 3 rooms designated for minor surgeries [6].

The hybrid methodology that combines the QFD (Quality Function Deployment), SERVQUAL and Fuzzy Logic (FL) tools emerges as a tool capable of helping in the planning of the quality of a product or service. This is possible, given that the QFD associated with SERVQUAL may be able to find gaps existing between the expectation and the perception of the activity performed, as to discern the characteristics of the service that are at a lower quality level, enabling the proposition of strategies capable of generating improvements and thereby delivering customer quality. Furthermore, the use of FL helps to avoid loss of information arising from human subjectivity, which contributes to making decisions more accurate [7, 8].

The present article proposes a hybrid QFD-fuzzy-SERVQUAL approach as a decision support tool for the quality planning of the HUOL surgical centre. In order to develop such approach it was necessary to take the following, it will be necessary to take the following methodological procedure: (1) elaborating and validating the SERVQUAL questionnaire; (2) identifying the main deficiencies of the surgical centre in the perception of the medical team through the application of SERVQUAL and fuzzy logic; (3) carrying out the construction of the ‘house of quality’, through the integration of the QFD-fuzzy-SERVQUAL tools; (4) performing the analysis of the engineering requirements through the ‘house of quality’ and fuzzy logic; and v) developing an action plan proposal for the surgical centre quality planning, resulting from the QFD-fuzzy-SERVQUAL integration.

### Quality function deployment—QFD

The Quality Function Deployment (QFD) methodology was initially developed with the objective of improving product development through user’s opinion. For this purpose, information is collected about the main needs of the client and then transformed into engineering criteria, which are used to solve the main demands related to the product in production [9].

**Fig. 1:**
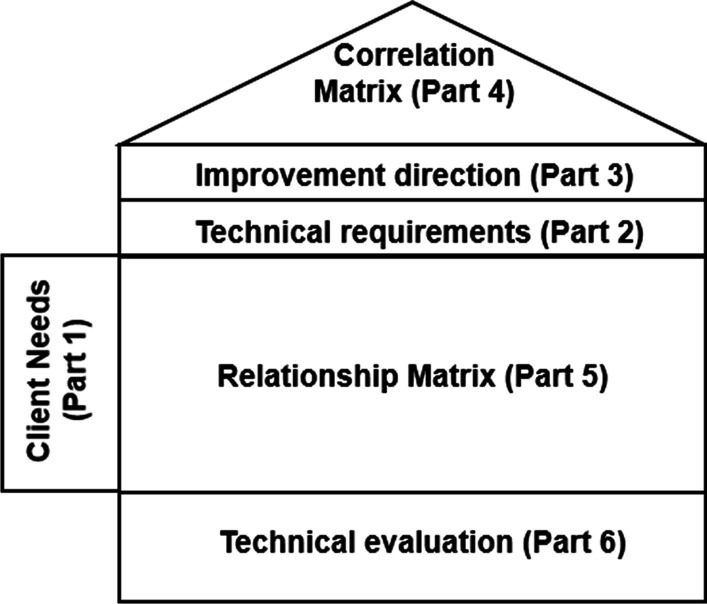
House of quality. *Source*: Adapted from Abdelsamad, Rushd and Tawfik [11]

One of the QFD key features is the conversion of qualitative requests into quantitative specifications, developing strategies that can improve the quality of planning and, ultimately, turning the QFD into a tool capable of assisting managerial decision making. Moreover, the QFD can be graphically represented as the “House of Quality” (HOQ) (Fig. 1), in which, through matrix analysis, it is possible to analyze the relationship between the customer demands and technical requirements and thereby propose improvements [10].

### SERVQUAL model

The SERVQUAL Model was developed by Parasuraman, Zeithaml and Berry [12] consisting of a system capable of measuring the difference between customer expectations and perceptions, referring to the perceived quality of a given product or service. SERVQUAL seeks to analyse in the object of study, dimensions related to tangibility, reliability, responsiveness, security, and empathy, to then identify needs and the greatest potential for improvement.

According to Büyüközkan et al. [13] the SERVQUAL model can evaluate service quality by analysing the data obtained by a questionnaire. Therefore, the evaluation of these data is performed using Eq. 1, where *P* and *E* represent, respectively, the perception and expectation of customers, with *Q* the size of the gap, that is, the present difference between the existing and the expected quality.

1 $$Q=P-E$$

Table 1 summarizes the criteria for service quality analysis.

**Table 1:** Quality service analysis criteria. *Source*: Adapted from Batista [14]

Relationship between perception (P) and expectation(E)	Service quality level
E > P	Service with quality below expectation
E < P	Service with quality above expectations
E = P	Service with neutral quality or within expected levels

### Fuzzy logic

Fuzzy logic, also known as fuzzy set theory, is a tool developed by researcher Zadeh [15] in a work called Fuzzy Sets, which is based on the classic concept of sets. However, it is distinguished by not working in a binary way, that is, it does not use only minimum (0) or maximum (1) values.

The FL development endeavours to portray, in an organized way, poorly defined elements and with a certain level of inconsistency, that is, inaccurate data. Thus, the FL uses a numerical range varying between 0 and 1 to represent different levels within a scale, being able to create a mathematical model to study phenomena with a certain level of uncertainty, but in a precise way [16, 17].

According to Ross [18], FL can be used for different types of applications, being considered a multidisciplinary tool capable of generating contributions in areas such as modelling of non-linear systems, pattern ordering, process management, as well as applications with qualitative data.

Another FL application is found in Büyüközkan, Çifçi and Güleryüz [13], to facilitate the inaccuracy estimation of human thought data. Given it is subjective, the authors claim that FL has the benefit of being able to represent the uncertainties of the human mind, as to assist decision makers in the interpretation and resolution of the problem studied.

### QFD-fuzzy

According to Raziei [1] the use of fuzzy logic associated with the QFD tools results in a better planning of the quality of a service; since, in the QFD methodology, it is necessary to carry out a series of analyses by a specialist involved in the development of the project. Hence, fuzzy logic helps to deal with the uncertainty of the data generated by the subjectivity present in each analyst's opinion.

Likewise, for Vaziri [19], the combination of QFD and fuzzy logic contributes to the improvement of the data analysis process and consequently improves experts’ decision-making. Hence, this hybrid approach may lead to advantages in the management of resources used in the project, for it is possible to prioritize the main actions to be performed, to improve the quality-of-service provision.

Furthermore, Saleh et al. [20] as well as Li et al. [21] point out that the combination of QFD and fuzzy logic works properly as a tool capable of assisting healthcare managers in activities or any decision-making process such as purchasing equipment, providing hospitals with a reduction in unnecessary expenses, as well as an increase in the quality of services due to better use of resources.

Finally, in Karsak and Dursun [22], QFD-fuzzy was applied as a group decision methodology, aiming to assist managers in the selection of suppliers for a private hospital in Istanbul; thus, the information obtained by the QFD is processed by the fuzzy logic, to avoid losing precious insights due to the subjectivity present in the opinion of the analysts that make up the decision group.

### Fuzzy logic promising applications

Literature reviews published in recent years indicated that Fuzzy Logic is opportune for the unification of ontologies involved in the decision-making process. Although it has presented such advantage since its inception, it has been the target of criticism from experts who consider it an approach that still lacks scientific support. Such statements have lost strength due to satisfactory applications of FL in sensor technology, electronics, and railways, for instance [23, 24].

Recent works have pointed out the application of Fuzzy Logic as a tool that, associated with techniques such as Artificial Intelligence, Machine Learning, Deep Learning and Multicriteria Decision Analysis, can contribute to mine opinions, in addition to supporting decisions and analyzing trends [25].

LF is identified as a multicriteria method by some literature reviews. Its integration with methods such as AHP (Analytic Hierarchy Process), TOPSIS (Techinique for Order of Preference by Similarity to Ideal Solution) and VIKOR (VIseKriterijumska Optimizacija I Kompromisno Resenje), for example, are accepted in high-impact scientific journals and contribute significantly for decision modelling [26–28].

## Methodology

### Universe and sample

HUOL’s SC is composed of a multidisciplinary team, such as doctors, nurses, nursing technicians, pharmacists, engineers, amongst other employees, who provide support for the correct functioning of the SC. However, due to standardization and technical knowledge, only doctors, nurses and nursing technicians were asked to participate in the application phase of the SERVQUAL questionnaire.

Once the sector was determined, the research began the stage of verifying the number of professionals who fall within the already established criteria. As a result of this filtering, a population of 147 individuals was defined, encompassing 64 doctors, 12 nurses and 71 nursing technicians and assistants. As for the questionnaire completion, it was determined that all professionals involved would have the same weight in the evaluations, given that no strictly technical knowledge was required from the medical team respondents. Participants gave written consent based on a semi-structured interview model developed and documented by the researchers in this study. The study was approved by the ethical committee of UFRN.

However, due to time and schedule limitations as well as the fact that the SC is a complex and biohazardous place, which makes the questionnaire difficult to apply, the research was carried through sampling. Therefore, based on expert opinion and scientific literature, it was decided to work with a sample that generates a 95% confidence level and a 5% margin of error.

### Data collection

Data collection was performed utilizing the SERVQUAL tool, which was applied at the HUOL’s SC. To carry out this procedure, HUOL's teaching, and research management was handed over all the requested documents. Furthermore, as it is a place with biological risk, they were properly attired, instructed and accompanied by a member of the medical team. The data collection lasted four days and occurred throughout the three shifts; only employees on their rest break were approached. Moreover, the respondents had the opportunity and were encouraged to ask questions regarding the survey during the time they were answering it.

### SERVQUAL application

To fill the questionnaire, the participants had to analyse all items according to three five-point scales, in which linguistic variables were established, so that members of the medical team could express their thoughts according to each situation.

The first scale is related to the employee's expectations regarding the quality of each item studied, considering the resources and possibilities existing at the time of the research. The second scale is related to the current perception that the member of the medical team has about the level of quality of each item studied.

Finally, the third scale aims to indicate the level of importance of each item, for the proper functioning of the SC, according to the respondent's opinion. Table 2 shows the three scales employed, their linguistic variables and their respective scores.

**Table 2:** Scales employed in SERVQUAL. *Source*: Adapted from Parasuraman, Zeithaml and Berry [29]

SCALE 1		SCALE 2		SCALE 3	
Expectation		Perception		Importance	
Linguistic variable	Assigned score	Linguistic variable	Assigned score	Linguistic variable	Assigned score
Very low	1	Too bad	1	Very low	1
Low	2	Bad	2	Low	2
Average	3	Average	3	Average	3
High	4	Good	4	High	4
Very high	5	Very good	5	Very high	5

With the utilization of scales 1 and 2, it is possible to identify the gaps between what the medical team considers to be ideal and what is being delivered, considering each item studied. Thus, it is feasible to obtain an overview of the current quality of the main points studied in the SC.

### SERVQUAL integration with the QFD-fuzzy approach

To carry out the integration amongst all the proposed approaches, a division was made into two phases, in which the first one will explain the necessary procedures to integrate SERVQUAL with the QFD tool. And in the second phase, the integration of FL into the study will be described.

#### Phase 1: integration of the QFD and SERVQUAL tools

**Fig. 2:**
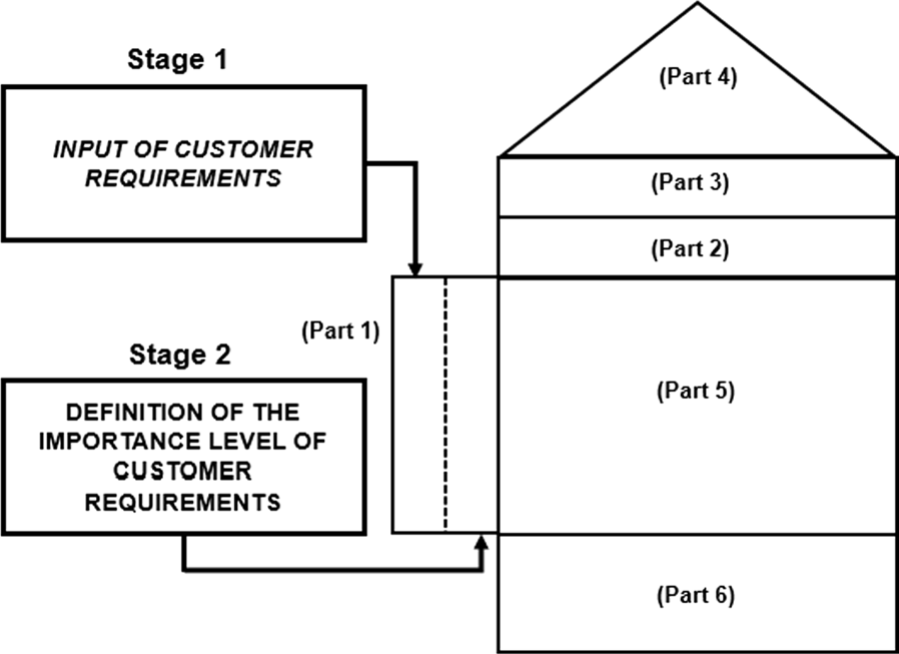
Schematic summary of the first phase of the integration of the studied methodologies

In phase 1, the integration model between the QFD and SERVQUAL tools is described. At this stage, the SERVQUAL methodology will act as a resource to provide inputs to the QFD, that is, it will provide the HOQ with the main requirements of the client and their respective levels of importance, based on the questionnaire applied to the SC medical team. Figure 2 depicts the steps explained in phase 1 of the integration between the methodologies utilized.

#### Phase 2: integration of the QFD and SERVQUAL tools with the FL

**Fig. 3:**
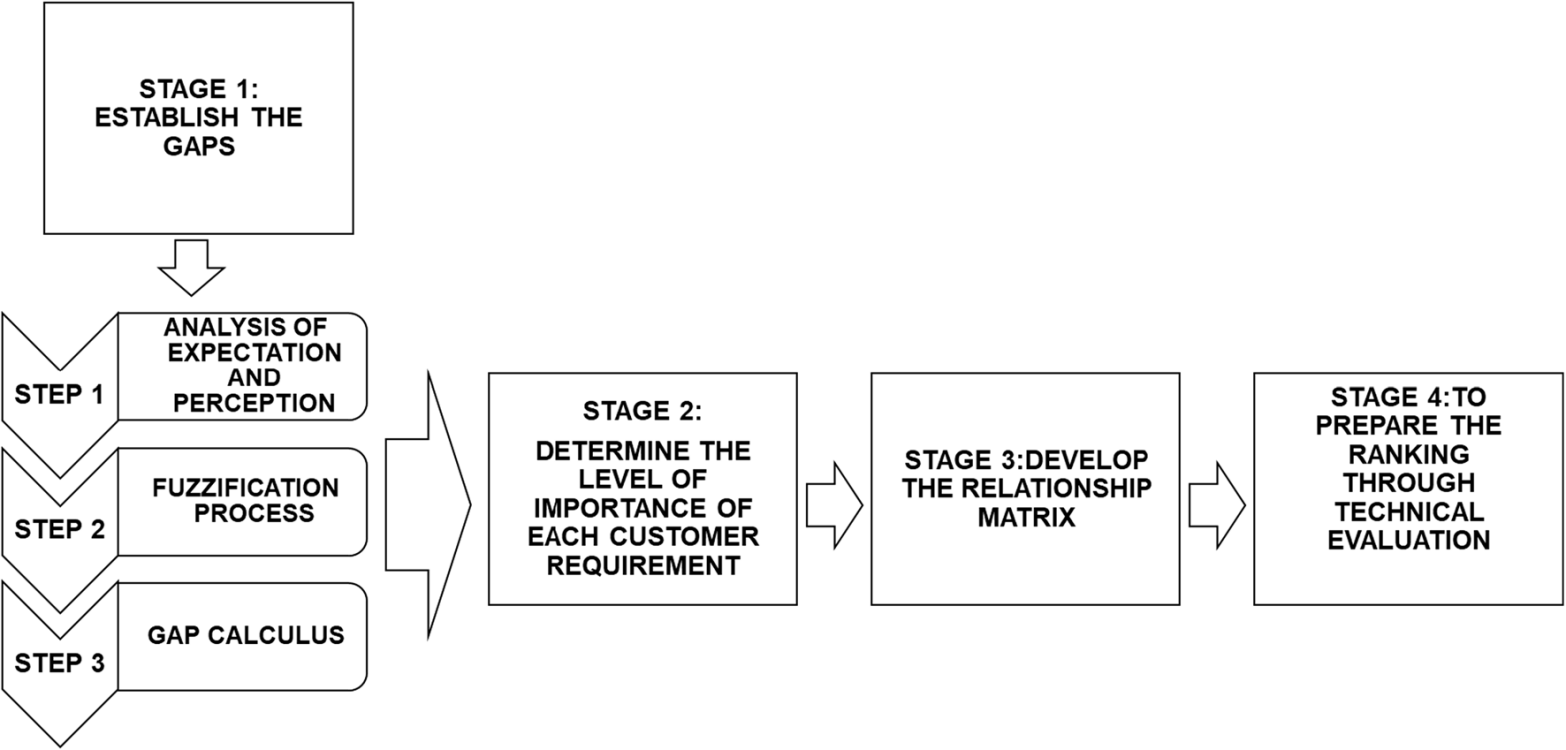
Schematic summary of the second phase of the studied methodologies integration

This phase main objective is to explain all stages of the FL integration with the combination of the QFD and SERVQUAL tools. Therefore, this arrangement aims to minimize the vagueness and imprecision existing in judgments involving linguistic variables. All operations performed will be done using the fuzzy theory established by Zadeh [15], in which triangular fuzzy numbers will be adopted due to their greater facility to perform mathematical operations [30–32]. Figure 3 summarizes phase 2 main stages in the current methodology, presenting how the integration and application of the utilized tools occurs.

*Stage 1: Utilizing the SERVQUAL-fuzzy combination to establish gaps in customer requirements (“customer voice”)*: In this stage, divided into three steps, the fuzzy numbers that will be used to represent the linguistic variables applied in the three different scales employed in the SERVQUAL questionnaire will be determined. To this end, the classic concept of fuzzy number and the fuzzification process established by Zadeh [15] will be adopted. Moreover, the operations used to define the gaps and the level of importance of each item in the questionnaire will be explained.

*Step 1: Scales 1 and 2 analysis:* As previously mentioned, the process of analyzing customers' expectations and perceptions regarding the 22 items of the SERVQUAL questionnaire will be carried out through a 5-point scale. In this case, each score will be represented by a linguistic variable, which in turn will be converted into a triangular fuzzy number through a process called fuzzification, which is explained in step 2.

*Step 2: Utilization of the triangular fuzzy number and the fuzzification process:* According to Wang [33] the fuzzification process consists of converting linguistic or numerical values, to the fuzzy universe. Therefore, to perform the fuzzification process, each linguistic variable corresponding to scales 1, 2 and 3 will be assigned a triangular fuzzy number, with an interval ranging from 0 to 1.

**Fig. 4:**
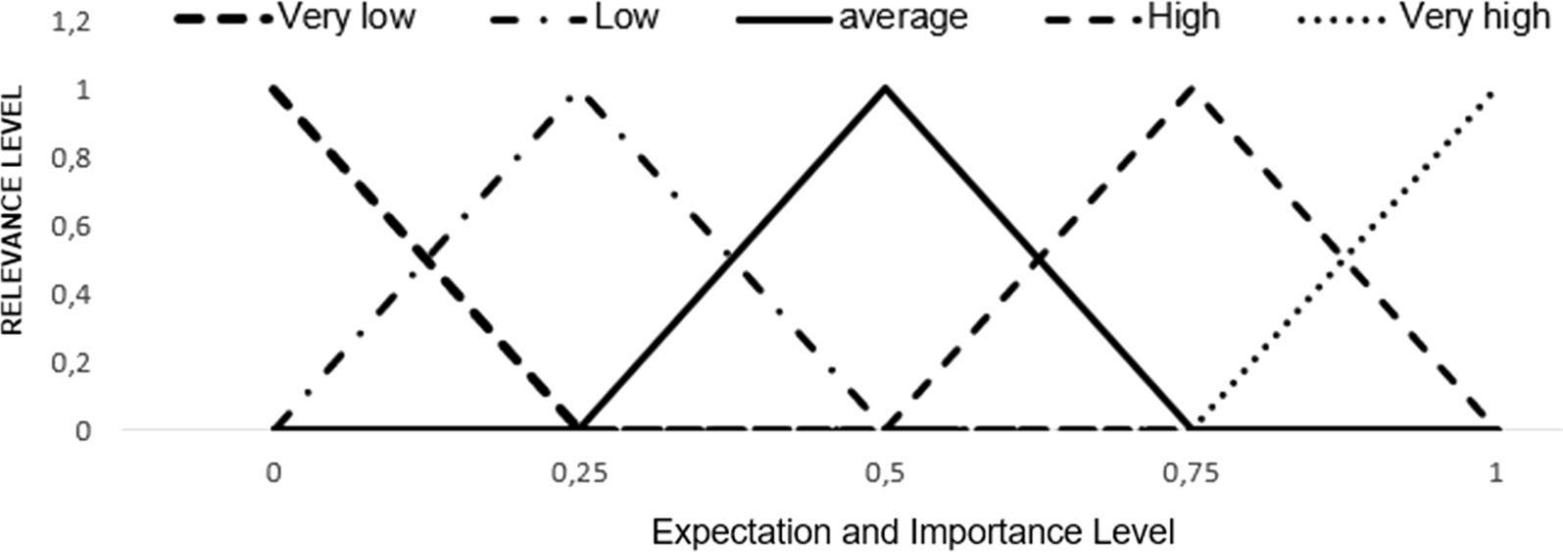
Graphical representation of fuzzy numbers corresponding to scales 1 and 3. *Source*: Adapted from Lima Junior, Osiro e Carpinetti [34]

**Fig. 5:**
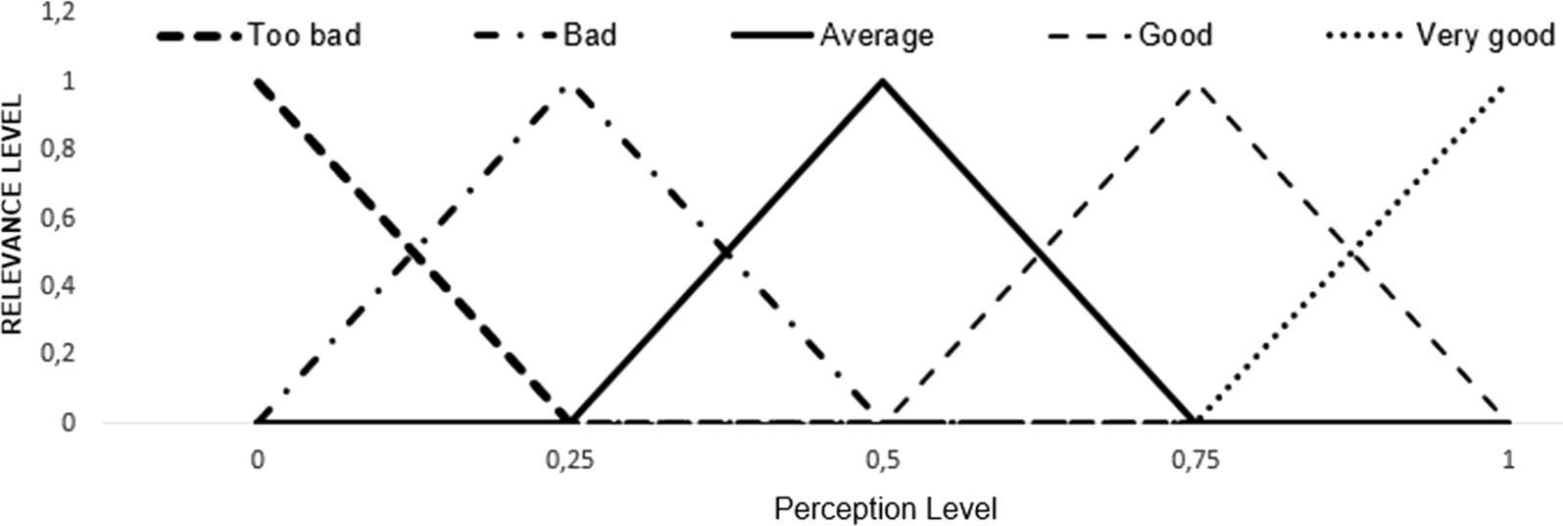
Graphical representation of fuzzy numbers corresponding to scale 2. *Source*: Adapted from Lima Junior, Osiro e Carpinetti [34]

Table 3 displays the linguistic variables used in each type of scale and their respective triangular fuzzy numbers, whilst Figs. 4 and 5 represent the graphic distribution for scales 1, 2 and 3.

**Table 3:** SERVQUAL questionnaire scales and their respective fuzzy numbers. *Source*: Adapted from Lima Junior, Osiro e Carpinetti [34]

Scale 1	Scale 2	Scale 3	Fuzzy number		
Expectation	Perception	Importance			
Linguistic variable	Linguistic variable	Linguistic variable	a	m	b
Very low	Too bad	Very low	0.0	0.0	0.25
Low	Bad	Low	0.0	0.25	0.5
Average	Average	Average	0.25	0.5	0.75
High	Good	High	0.5	0.75	1
Very high	Very good	Very high	0.75	1	1

*Step 3: Using operations to calculate the gap:* In this step, three operations are used. The first has the function of calculating the arithmetic mean of the score obtained in each of the 22 items studied, considering the assessment of all respondents, using scales 1 and 2. After that, a second fuzzy operation is performed to determine the existing gaps in all items, through the difference between scales 1 and 2. Finally, a third equation is used to perform the process of defuzzification of the triangular numbers obtained in the gap calculation.

To calculate the average between triangular fuzzy numbers, Eqs. 2 and 3 will be used, which in turn were applied based on Buckey [35], Chen [36], and Cho, Kim and Kwak [37], where ${\tilde{E }}_{i}$ e ${\tilde{P }}_{i}$ represent, respectively, the expectation and the perception in the process of the average evaluation of each item of the questionnaire, with k = 1, 2, 3 … total evaluators and i = 1, 2, 3… total items assessed by the SERVQUAL questionnaire.

2 $${\tilde{E }}_{i}= \frac{1}{k}\left[{\tilde{E }}_{i}^{1}\left(+\right){\tilde{E }}_{i}^{2}\left(+\right)\dots \left(+\right){\tilde{E }}_{i}^{k}\right]$$

3 $${\tilde{P }}_{i}= \frac{1}{k}\left[{\tilde{P }}_{i}^{1}\left(+\right){\tilde{P }}_{i}^{2}\left(+\right)\dots \left(+\right){\tilde{P }}_{i}^{k}\right]$$

Once the fuzzy operation proposed by Eqs. 2 and 3 was performed, a second operation is also performed to calculate the gap between the fuzzy numbers corresponding to each of the 22 analysed items. To that end, the fuzzy operator described in Eq. 4 is utilized. Therefore, the values for each average assessment of expectation and perception will be represented, respectively, by fuzzy numbers described as ${\tilde{E }}_{i}=({E}_{a},{E}_{m},{E}_{b})$ e ${\tilde{P }}_{i}=({P}_{a},{P}_{m},{P}_{b})$ [38–40].

4 $${GAP= \tilde{P }}_{i}- {\tilde{E }}_{i}=\left({P}_{a}-{E}_{b}, {P}_{m}-{E}_{m}, {P}_{b}-{E}_{a}\right)=\left({A}_{a}, {B}_{m}, {C}_{b}\right)$$

Subsequently, the third operation consists of applying to all gaps obtained by Eq. 4 the defuzzification process proposed by Eq. 5, which, as already mentioned, consists of converting a fuzzy number into a real value, also called a crisp number [41].

For this purpose, the operator proposed in the work of Chen and Hsieh [42] as well as Behdioğlu, Acar and Burhan [43] will be utilized in the defuzzification of values obtained through SERVQUAL

5 $$D= \frac{{A}_{a}+ {4B}_{m}+{C}_{b}}{6}$$

Thereby, it will be possible to obtain the gap value of each item studied, to identify and represent a real number that adequately portrays the client's feeling and the level of quality of the studied object.

### Stage 2: Utilizing fuzzy logic to determine the level of importance for each customer requirement.

Once stage 1 is complete, the process of defining the level of importance of each item will begin. For this purpose, Eq. 6 described below will be used, where ${\tilde{w }}_{i}$ represents the average rating of each item on scale 3, with k = 1, 2, 3… total evaluators and i = 1, 2, 3…total items assessed. This will define the triangular fuzzy number equivalent to the average importance of each item analysed in the SERVQUAL questionnaire. However, only fuzzy numbers that have a gap with a crisp value less than or equal to the average of all items will be used in the next step.

6 $${\tilde{W }}_{i}= \frac{1}{k}\left[{W}_{i}^{1}\left(+\right){W}_{i}^{2}\left(+\right)\dots \left(+\right){W}_{i}^{k}\right]$$

### Stage 3: Utilizing fuzzy logic in the relationship matrix elaboration:

In this stage, the analysis of the relationship between each customer's requirement weights (${\tilde{W }}_{i}$) with engineering requirements (${\tilde{H }}_{j}$) will be carried out. For that purpose, each element ${\tilde{R }}_{ij}$,—the existing relationship in the matrix—will be analyzed by experts based on Table 2 as to assign a relationship level represented by symbols and associated with linguistic variables, which in turn will be translated into triangular fuzzy numbers. Equation 7 describes the average assessment of the relationship level assigned by each specialist, with k being the total participating specialists, where $i=1, 2, 3\dots n$ and $j=1, 2, 3\dots m$, that is, the elements $n$ and $m$ represent, respectively, the total of ${\tilde{W }}_{i}$ and ${\tilde{H }}_{j}$ present in the study. Table 4 contributes to the understanding of the fuzzy sets applied in this study.

**Table 4:** Relationship levels, their respective symbols, and fuzzy numbers. *Source*: Adapted from Kargari [44]

Relationship level	Linguistic variable symbol	Fuzzy numbers		
		a	m	b
Very low	VL	0.0	0.0	0.25
Low	L	0.0	0.25	0.5
Average	A	0.25	0.5	0.75
Strong	S	0.5	0.75	1.0
Very strong	VS	0.75	1.0	1.0
Non-existent	–	–	–	–

7 $${\tilde{R }}_{ij}= \frac{1}{k}\left[{R}_{ij}^{1}\left(+\right){R}_{ij}^{2}\left(+\right)\dots \left(+\right){R}_{ij}^{k}\right]$$

**Fig. 6:**
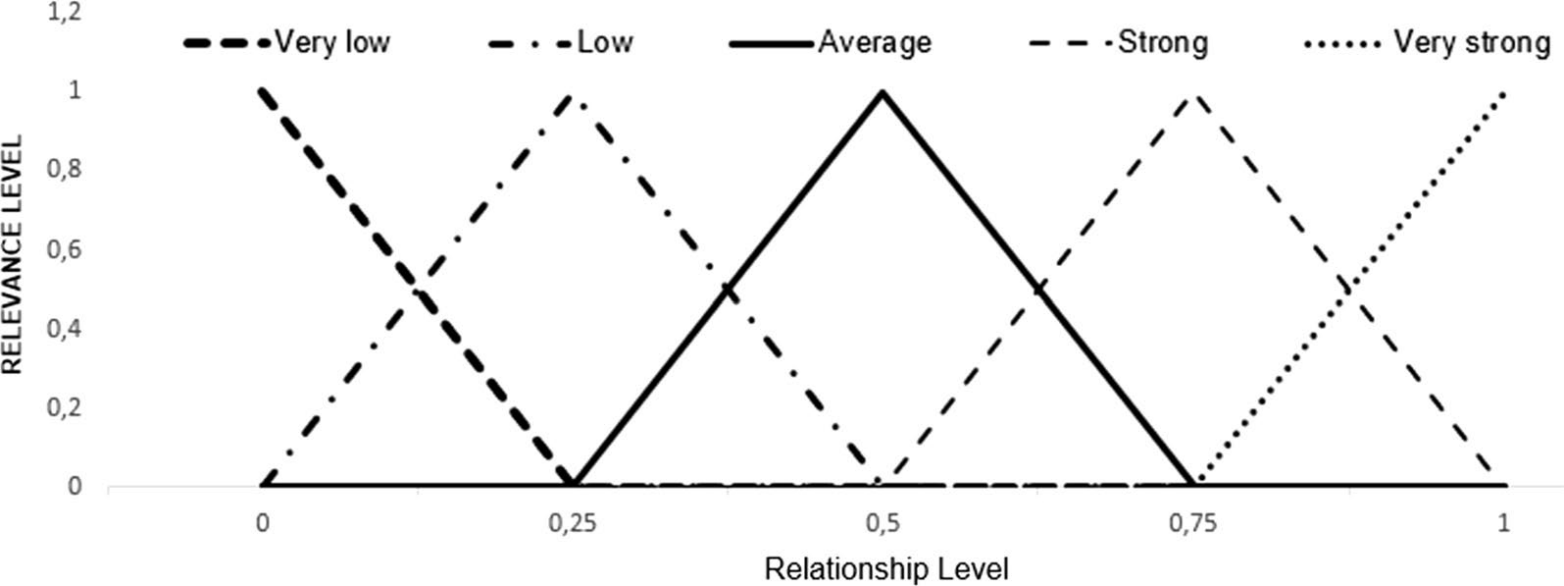
Graphical representation of the fuzzy numbers corresponding to Table 2. *Source*: Adaptaded from Kargari [44]

Finally, Table 2 is represented in Fig. 6, where it reproduces the triangular fuzzy numbers and their respective linguistic variables.

### Stage 4: Utilizing fuzzy logic in the technical assessment to determine the relative importance of each engineering requirement.

*Stage 5: Consists of exposing the process of calculating the relative importance of each* ${\tilde{H }}_{j}$ *present in the HOQ. For that, it will be utilized a relationship between the value of each* ${\tilde{W }}_{i}$ *and* ${\tilde{R }}_{ij}$ *.*

According to Bottani [45] the relationship between ${\tilde{W }}_{i}$ and ${\tilde{H }}_{j}$ can be represented as ${\tilde{R }}_{ij}$, in which Eq. 8 is used to calculate the relative importance of each ${\tilde{H }}_{j}$, which in turn is represented by ${\tilde{IR }}_{j}$.

8 $${\tilde{IR }}_{j}=\sum_{i=1}^{n}{(\tilde{W }}_{i}).\left({\tilde{R }}_{ij}\right) , j=1, 2, \dots ,m$$

## Results

### Quality gaps

This specific stage has as key objectives to identify and analyze the gaps of quality referring to the 22 items studied. To achieve these goals, it was necessary to assess the expectations and perceptions of the SC medical team, using the SERVQUAL questionnaire. Table 5 represents the quality gaps related to each item studied.

**Table 5:** Quality gaps representation through fuzzy and crisp numbers

Dimensions	ITENS	FUZZY numbers			Crisp numbers
		a	m	b	
Tangibility	Item 1	− 0.65	− 0.28	0.20	− 0.26
	Item 2	− 0.55	− 0.20	0.28	− 0.18
	Item 3	− 0.47	− 0.13	0.30	− 0.12
	Item 4	− 0.61	− 0.24	0.23	− 0.23
	Item 5	− 0.53	− 0.19	0.26	− 0.17
Reliability	Item 6	− 0.51	− 0.17	0.27	− 0.15
	Item 7	− 0.59	− 0.23	0.24	− 0.21
	Item 8	− 0.46	− 0.15	0.27	− 0.13
	Item 9	− 0.55	− 0.20	0.25	− 0.19
	Item 10	− 0.66	− 0.32	0.17	− 0.29
Responsiveness	Item 11	− 0.65	− 0.28	0.20	− 0.26
	Item 12	− 0.53	− 0.23	0.20	− 0.21
	Item 13	− 0.55	− 0.24	0.20	− 0.22
Security	Item 14	− 0.76	− 0.47	0.01	− 0.44
	Item 15	− 0.77	− 0.49	− 0.01	− 0.45
	Item 16	− 0.74	− 0.45	0.03	− 0.42
	Item 17	− 0.52	− 0.21	0.24	− 0.18
	Item 18	− 0.57	− 0.24	0.21	− 0.22
	Item 19	− 0.44	− 0.12	0.29	− 0.10
Empathy	Item 20	− 0.53	− 0.20	0.24	− 0.18
	Item 21	− 0.60	− 0.28	0.18	− 0.26
	Item 22	− 0.25	− 0.18	0.27	− 0.15

**Fig. 7:**
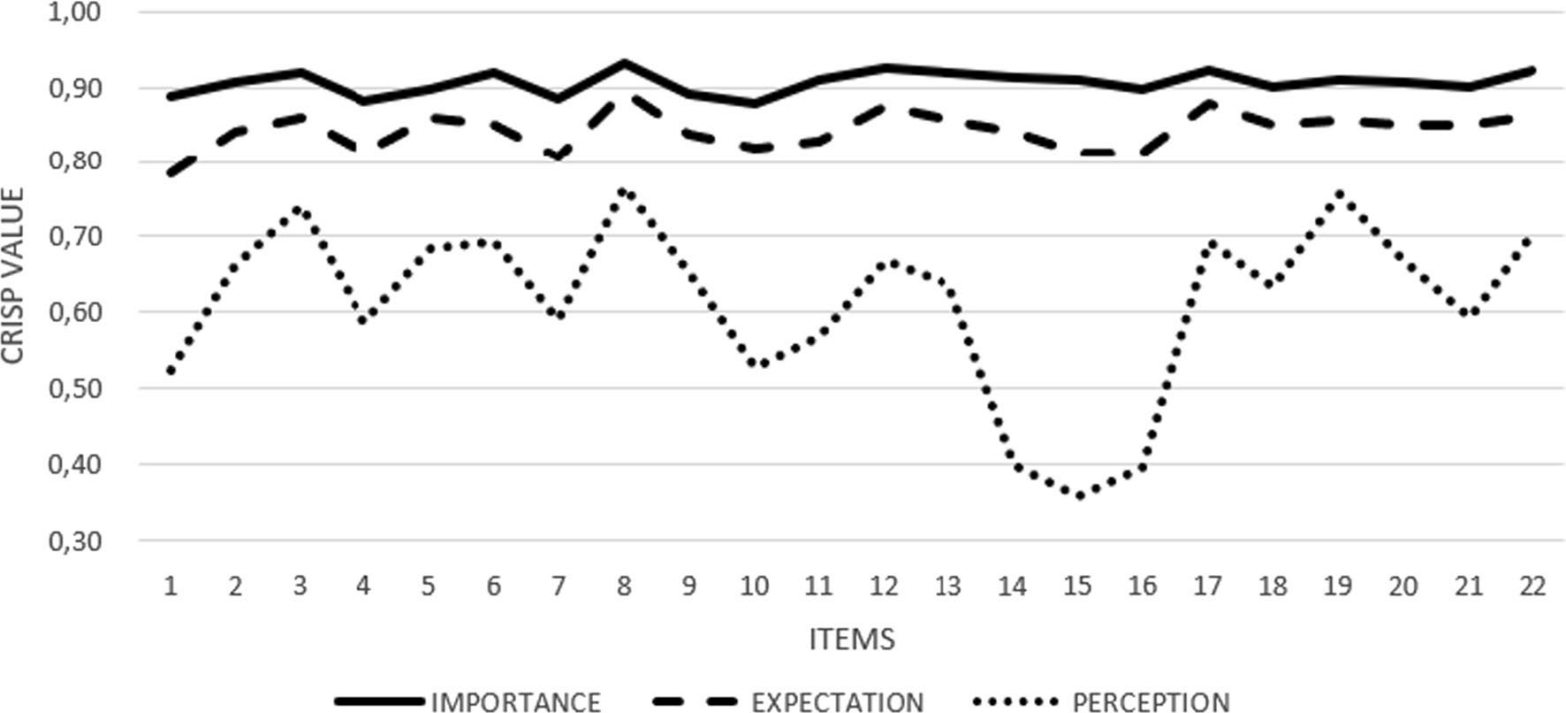
Graphical representation of the quality gaps

In short, Fig. 7 displays a graphic representation of the general levels of expectation, perception, and importance of all the items studied, in which the space between the dotted lines symbolizes the gaps in the quality of the SC.

### Defining the “Voice of the Customer – VOC”

Once all gaps have been calculated, it is feasible to define which items will be used in the HOQ, thus forming the “voice of the customer”. Thus, it was established the utilization of the items that only have a gap with a crisp value less than or equal to − 0.23, given that this number represents the average of the gaps of all items. Table 6 presents the appointed items, displaying their placement in the largest gaps ranking as well as their respective size and description.

**Table 6:** Definition of “customer's voice”

Items	GAP	Ranking	Dimension	Item description
Item 15	− 0.45	1	Security	Training for accident situations (rapid evacuation, fire, explosion, etc.)
Item 14	− 0.44	2	Security	Contingency plan for disaster situations (rapid evacuation, fire, explosion, etc.)
Item 16	− 0.42	3	Security	The physical structure is adequate for the safety measures in place (Correct signaling, operation of generators, presence of anti-panic door, operation of fire-fighting equipment)
Item 10	− 0.29	4	Reliability	Surgical planning and schedule
Item 1	− 0.26	5	Tangibility	General physical structure (hydraulic, electrical, facilities, furniture, etc.)
Item 11	− 0.26	6	Responsiveness	Operating rooms demand-response capacity
Item 21	− 0.26	7	Empathy	Employees fully understand the need to assist other team members
Item 4	− 0.23	8	Tangibility	Technical team (engineering, administration, maintenance, and hygiene)

### House of Quality (HOQ) assessment

At this stage, the relationship between Customer Needs (CN) and Project Requirements (PR) will be assessed. (STANDARDIZING THE NOMENCLATURE) Thus, it will be feasible to study how strong the link between these requirements really is. Moreover, it will be feasible to define the contribution level of each PR to achieve the quality defined by the “voice of the customer”.

The relationship matrix was filled out based on three expert’s opinion, who are from the medical, engineering, and occupational safety areas, given that the needs established by the “voice of the customer” require multidisciplinary knowledge to be associated with the PR. Consequently, each specialist was responsible for evaluating only the cells corresponding to the needs of the customers related to their area of specialization. Moreover, they were also responsible for translating the “voice of the customer” into PRs. Table 7 shows all the PRs obtained by the specialists, from the CNs.

**Table 7:** Project requirements

Project requirements (PR)	Description
PR1	Creation of an obligatory training agenda and/or training against casualties (quick evacuation, fire, explosion, etc.)
PR2	Creation of a contingency plan against casualties (quick evacuation, fire, explosion, lack of water or energy, etc.)
PR3	Adoption of technical safety standards determined by the fire department
PR4	Integrated management system (which allows integration between stock data, human resources and the list of registered surgeries)
PR5	Adoption of technical standards relating to planning, programming, elaboration and evaluation of physical projects in health care establishments
PR6	Monitoring of surgery rooms operating dynamics
PR7	Shared management (allowing all members of the team to participate in decisions)
PR8	Training the technical team (engineering, administration, maintenance and hygienization)

It is worth pointing out that the experts’ opinion was initially represented by real numbers that have been attributed based on linguistic variables. Then, all values were converted into triangular fuzzy numbers, according to the scale shown in Table 2. Hence, Table 8 presents a relationship matrix containing the fuzzy representation of all relationships, as well as the relative importance of each PR.

**Table 8:** Fuzzy relationship matrix

	Level of importance			PR 1			PR 2			PR 3			PR 4			PR 5			PR 6			PR 7			PR 8		
CN 1	0.69	0.94	0.98	0.75	1.0	1.00	0.75	1.0	1.00	0.75	1.00	1.00	0.00	0.0	0.00	0.00	0.00	0.00	0.00	0.00	0.00	0.50	0.75	1.00	0.75	1.00	1.0
CN 2	0.70	0.95	0.99	0.75	1.0	1.00	0.75	1.0	1.00	0.75	1.00	1.00	0.00	0.0	0.00	0.25	0.50	0.75	0.00	0.00	0.00	0.50	0.75	1.00	0.75	1.00	1.0
CN 3	0.69	0.93	0.98	0.75	1.0	1.00	0.75	1.0	1.00	0.75	1.00	1.00	0.00	0.0	0.00	0.50	0.75	1.00	0.00	0.00	0.25	0.50	0.75	1.00	0.75	1.00	1.0
CN 4	0.66	0.91	0.97	0.00	0.0	0.00	0.00	0.0	0.25	0.00	0.00	0.25	0.75	1.0	1.00	0.75	1.00	1.00	0.75	1.00	1.00	0.75	1.00	1.00	0.50	0.75	1.0
CN 5	0.67	0.92	0.97	0.00	0.0	0.00	0.25	0.5	0.75	0.50	0.75	1.00	0.00	0.0	0.25	0.25	0.50	0.75	0.25	0.50	0.75	0.00	0.00	0.25	0.00	0.25	0.5
CN 6	0.69	0.94	0.99	0.00	0.0	0.25	0.00	0.0	0.25	0.00	0.00	0.25	0.75	1.0	1.00	0.75	1.00	1.00	0.75	1.00	1.00	0.75	1.00	1.00	0.75	1.00	1.0
CN 7	0.68	0.93	0.99	0.25	0.5	0.75	0.25	0.5	0.75	0.25	0.50	0.75	0.75	1.0	1.00	0.50	0.75	1.00	0.75	1.00	1.00	0.75	1.00	1.00	0.75	1.00	1.0
CN 8	0.66	0.91	0.98	0.00	0.0	0.00	0.25	0.5	0.75	0.00	0.25	0.50	0.25	0.5	0.75	0.00	0.25	0.50	0.50	0.75	1.00	0.25	0.50	0.75	0.75	1.00	1.0
Relative importance (Fuzzy)				1.7	3.3	3.9	2.1	4.2	5.6	2.1	4.2	5.6	1.7	3.2	3.9	2.0	4.4	5.9	2.0	3.9	4.9	2.7	5.4	6.9	3.4	6.5	7.4
Relative importance (Crisp)				3.1			4.1			4.1			3.1			4.3			3.8			5.2			6.1		
Relative importance (%)				9.29			12.11			12.12			9.17			12.62			11.17			15.32			18.20		
Ranking				7			5			4			8			3			6			2			1		

Through the relationship matrix analysis (Table 8), it is feasible to verify that the PR that have the greatest relative importance is correlated to the technical teams training (6.1), in which the requirements related to shared management were also highlighted (5.2) and adoption of technical standards related to planning, programming, development and evaluation of physical projects in health care establishments (4.3).

**Fig. 8:**
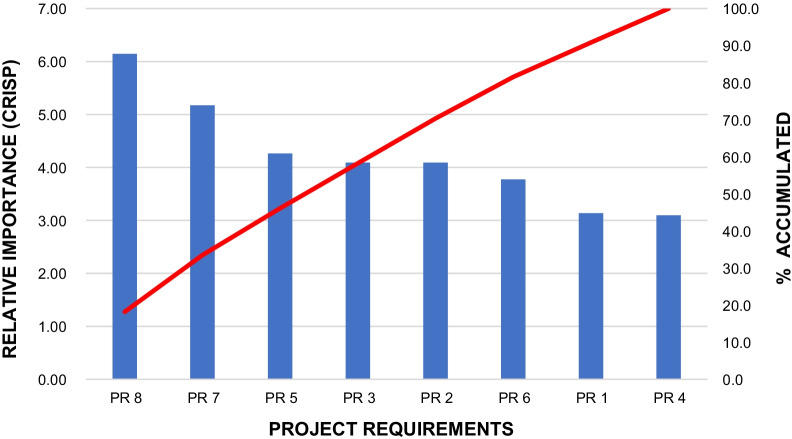
Representation of the relative importance of the design requirements using the Pareto diagram

Furthermore, it is possible to observe in Fig. 8 the ranking of the relative importance of each PR being represented in the histogram by crisp values, where the accumulated value of the relative importance of the PR is also shown by the red line. Thus, it is possible to define which actions should initially be prioritized, aiming at customer satisfaction.

Once the PR ranking with the highest crisp values has already been obtained (Table 8), it is feasible to prioritize which actions should be taken by the top management of the HUOL. Hence, it is relevant to highlight which conclusions can be taken from the main PR, as to assist managers throughout the action plans preparation.

The PR8—TRAININING THE TECHNICAL TEAM (ENGINEERING, ADMINISTRATION, MAINTENANCE AND HYGIENIZATION) is the most relevant due to its influence in addressing the “voice of the customer”. Thus, top management should be guided to study ways to improve the technical teams training.

Second in the ranking, PR7 – SHARED MANAGEMENT (ALLOWING ALL MEMBERS OF THE TEAM TO PARTICIPATE IN DECISIONS) reveals that top management needs to discuss with the main SCs managers, ways to improve the participation of all HUOL employees in the decision-making processes related to the SC.

Third in the ranking, PR5—ADOPTION OF TECHNICAL STANDARDS RELATING TO PLANNING, PROGRAMMING, ELABORATION AND EVALUATION OF PHYSICAL PROJECTS IN HEALTH CARE ESTABLISHMENTS demonstrates that there is a need to assess whether the technical standards in question are being properly applied, since this PR can improve the SC quality.

Furthermore, it is also important to highlight the requirements related to PR3—ADOPTION OF TECHNICAL SAFETY STANDARDS DETERMINED BY THE FIRE DEPARTMENT as well as PR2—CREATION OF A CONTINGENCY PLAN AGAINST CASUALTIES (QUICK EVACUATION, FIRE, EXPLOSION, LACK OF WATER OR ENERGY, ETC.); in which they point to the need for investments in occupational safety engineering, either through training against accidents or by adapting the SC's infrastructure to safety standards.

## Conclusion

The research succeeded in cataloguing the perception of the actors involved in the planning of Clinical Engineering operations at the HUOL Surgical Centre, directing intervention priorities according to systematizations supported by Decision Theory.

The economy precept, understood as a relevant practice in the Brazilian public management morality, was evidenced from this study and contributes for HUOL to continue complying with its commitment to formal aspects aligned with governance and regulatory bodies.

The present work limitations derive mainly from its quali-quantitative approach, as it may not reliably represent the respondent's perception. The SERVQUAL technique itself can cause biases when compared to statistically validated protocols, which puts into question whether there would be other tools more suitable for service planning than the one applied in this study to obtain the voice of the customer step in the QFD-fuzzy.

As a suggestion for future research, the authors consider the possibility that a structural equation model should be developed to identify, from a conceptual model, the factors considered priority (critical) in the planning of Clinical Engineering operations in Surgical Centres throughout the network of university hospitals managed by the Brazilian Hospital Services Company (EBSERH) presenting scenarios of financial implications impacted by this model. Through this network of hospitals it is possible to apply techniques such as Opinion Mining exploring new discussions about Fuzzy Logic.

## Data Availability

The data are with the corresponding author (Jurandir Barreto Galdino Junior) who makes them available upon request.

## References

[1] Rezaei T, Ghahramanian A, Abdullahzaed F, Sheikhalipour Z, Asghari-Jafarabadi M, Fadaei Z (2018). Service quality gaps in the provision of care to surgical patients: a cross-sectional study in the Northwest of Iran. J Caring Sci.

[2] Bidassie B, Gunnar W, Starr L, Van Buskirk G, Warner L, Anckaitis C, Howard A (2018). Data-driven process to improve VA surgical flow. Int J Health Care Qual Assur.

[3] Nazif, H. Operating room surgery scheduling with fuzzy surgery durations using a metaheuristic approach. Adv Oper Res. 2018. 10.1155/2018/8637598.

[4] Nasiri MM, Shakouhi F, Jolai F (2019). A fuzzy robust stochastic mathematical programming approach for multi-objective scheduling of the surgical cases. Opsearch.

[5] Barbagallo S, Corradi L, de Ville de Goyet J, Iannucci M, Porro I, Rosso N, Tanfani E, Testi A (2015). Optimization and planning of operating theatre activities: an original definition of pathways and process modeling. BMC Med Inform Decis Mak.

[6] Empresa Brasileira de Serviços Hospitalares. Institucional [internet]. Brasília: EBSERH; [cited 2020 Jan 06]. Avaliable from: https://www.gov.br/ebserh/pt-br/hospitais-universitarios/regiao-nordeste/huol-ufrn/acesso-a-informacao/institucional

[7] Priyono A, Yulita A (2017). Improving service quality of hospital front office using an integrated Kano model and quality function deployment. Intangible Capital.

[8] Raziei Z, Torabi AS, Tabrizian S, Zahiri B (2018). A hybrid GDM-SERVQUAL-QFD approach for service quality assessment in hospitals. Eng Manag J.

[9] Akao Y (1990). Quality function deployment—QFD: integrating customer requirements into product design.

[10] Jaffray DA, Easty T. Erratum: world congress on medical physics and biomedical engineering. In: Jaffray D. World Congress on Medical Physics and Biomedical Engineering. Toronto: Springer; 2015. p. 7–12. 10.1007/978-3-319-19387-8_427

[11] Yassin A, Muhammad R, Bassel T. Functional and spatial design of emergency departments using quality function deployment. J Healthcare Eng. 2018; 2018:1–7. 10.1155/2018/9281396 10.1155/2018/9281396 PMC6311852 30651949

[12] Parasuraman A, Zeithaml VA, Berry LL (1985). A conceptual model of service quality and its implications for future research. J Mark.

[13] Büyükozkan G, Çifçi G, Güleryüz S (2011). Strategic analysis of healthcare servisse quality using fuzzy AHP methodology. Exp Syst Appl.

[14] Batista DA. O Uso da Abordagem Fuzzy para a Integração das Ferramentas QFD e SERVQUAL em Serviços de Saúde[dissertation]. Pernambuco: Universidade Federal de Pernambuco; 2013. https://repositorio.ufpe.br/handle/123456789/12938

[15] Zadeh LA (1965). Fuzzy sets. Inf Control.

[16] Zimmermann HJ. Fuzzy set theory and its application. 3rd ed. Kluwer Academic Publishers, Boston; 1996.

[17] Shaw IS, Simõe MG. Controle e Modelagem Nebulosa. São Paulo: editora Edgard Blucher; 1999. p 165.

[18] Ross TJ (2004). Fuzzy logic with engineering applications.

[19] Vaziri J, Beheshtinia MA (2016). A holistic fuzzy approach to create competitive advantage via quality management in services industry (case study: life-insurance services). Manag Decis.

[20] Saleh N, Sharawi AA, Abdel Wahed M, Balestra G (2015). A Conceptual Priority Index for Purchasing Medical Equipment in Hospitals. J Clin Eng.

[21] Lin LZ, Chen WC, Chang TJ (2011). Using FQFD to analyze island accommodation management in fuzzy linguistic preferences. Exp Syst Appl.

[22] Karsak EE, Dursun M (2015). An integrated fuzzy MCDM approach for supplier evaluation and selection. Comput Ind Eng.

[23] Kahraman C, Öztayşi B, Çevik Onar S. A comprehensive literature review of 50 years of fuzzy set theory. Int J Comput Intell Syst. 2016;9(May):3–24.

[24] Jiang Y, Yang C, Ma H. A review of fuzzy logic and neural network based intelligent control design for discrete-time systems. Discret Dyn Nat Soc. 2016;2016.

[25] Serrano-Guerrero J, Romero FP, Olivas JA. Fuzzy logic applied to opinion mining: a review. Knowledge-Based Syst. 2021;222:107018. 10.1016/j.knosys.2021.107018.

[26] Mendoza GA, Martins H (2006). Multi-criteria decision analysis in natural resource management: a critical review of methods and new modelling paradigms. For Ecol Manag.

[27] Frazão TDC, Camilo DGG, Cabral ELS, Souza RP (2018). Multicriteria decision analysis (MCDA) in health care: a systematic review of the main characteristics and methodological steps. BMC Med Inform Decis Mak.

[28] Opricovic S (2011). Fuzzy VIKOR with an application to water resources planning. Exp Syst Appl.

[29] Parasuraman A, Zeithaml VA, Berry LL (1988). SERVQUAL: a multipleitem scale for measuring consumer perceptions of service quality. J Retail.

[30] Guinta LR, Praizler NC (1993). Manual de QFD—O uso de equipes para solucionar problemas e satisfazer clientes pelo desdobramento da função qualidade.

[31] Chou CC, Liu LJ, Huang SF, Yih JM, Han TC. An evaluation of airline service quality using the fuzzy weighted SERVQUAL method. Appl Soft Comput. 2011;11(2):2117–2128. 10.1016/j.asoc.2010.07.010

[32] Prascevic Z, Prascevic N (2013). One modification of fuzzy TOPSIS method. J Model Manag.

[33] Wang P (1996). The interpretation of fuzziness. IEEE Trans Syst Man Cybern Syst.

[34] Junior FRL, Osiro L, Carpinetti LCR (2014). A comparison between fuzzy AHP and fuzzy TOPSIS methods to supplier selection. Appl Soft Comput.

[35] Buckley JJ (1985). Ranking alternatives using fuzzy numbers. Fuzzy Sets Syst.

[36] Chen CT (2001). A fuzzy approach to select the location of the distribution center. Fuzzy Sets Syst.

[37] Cho IJ, Kim YJ, Kwak C (2015). Application of SERVQUAL and fuzzy quality function deployment to service improvement in service centres of electronics companies. Total Qual Manag Bus Excell.

[38] Kaufman A, Gupta MM (1991). Introduction to fuzzy arithmetic.

[39] Hu HY, Lee YC, Yen TM (2010). Service quality gaps analysis based on Fuzzy linguistic SERVQUAL with a case study in hospital out-patient services. Total Qual Manag.

[40] Beheshtinia MA, Azad FM (2017). A fuzzy QFD approach using SERVQUAL and Kano models under budget constraint for hotel services. Total Qual Manag Bus Excell.

[41] Dobrosielski WT, Czerniak JM, Zarzycki H, Szczepański J. Fuzzy numbers applied to a heat furnace control. In: Studies in fuzziness and soft computing. 2017. p. 269–88.

[42] Chen SH, Hsieh CH. Graded mean integration representation of generalized fuzzy number. In: Proceedings of Sixth Conference on Fuzzy Theory and its Application; 1998, Taiwan, Republic of China. Chinese Fuzzy Systems Association, 1998.

[43] Behdioğlu S, Acar E, Burhan HA (2017). Evaluating service quality by fuzzy SERVQUAL: a case study in a physiotherapy and rehabilitation hospital. Total Qual Manag Bus Excell.

[44] Kargari M (2018). Ranking of performance assessment measures at tehran hotel by combining DEMATEL, ANP, and SERVQUAL models under fuzzy condition. Math Probl Eng.

[45] Bottani E (2009). A fuzzy QFD approach to achieve agility. Int J Prod Econ.

